# Seladin-1 expression is regulated by promoter methylation in adrenal cancer

**DOI:** 10.1186/1471-2407-10-201

**Published:** 2010-05-13

**Authors:** Lisa Simi, Francesca Malentacchi, Paola Luciani, Stefania Gelmini, Cristiana Deledda, Rosaria Arvia, Massimo Mannelli, Alessandro Peri, Claudio Orlando

**Affiliations:** 1 Clinical Biochemistry, Department of Clinical Physiopathology, University of Florence, viale Pieraccini 6, Florence, 50139 Italy; 2 Endocrinology Units, Department of Clinical Physiopathology, University of Florence, viale Pieraccini 6, Florence, 50139 Italy

## Abstract

**Background:**

Seladin-1 overexpression exerts a protective mechanism against apoptosis. Seladin-1 mRNA is variably expressed in normal human tissues. Adrenal glands show the highest levels of seladin-1 expression, which are significantly reduced in adrenal carcinomas (ACC). Since up to now seladin-1 mutations were not described, we investigated whether promoter methylation could account for the down-regulation of seladin-1 expression in ACC.

**Methods:**

A methylation sensitive site was identified in the seladin-1 gene. We treated DNA extracted from two ACC cell lines (H295R and SW13) with the demethylating agent 5-Aza-2-deoxycytidine (5-Aza). Furthermore, to evaluate the presence of an epigenetic regulation also 'in vivo', seladin-1 methylation and its mRNA expression were measured in 9 ACC and in 5 normal adrenal glands.

**Results:**

The treatment of cell lines with 5-Aza induced a significant increase of seladin-1 mRNA expression in H295R (fold increase, F.I. = 1.8; p = 0.02) and SW13 (F.I. = 2.9; p = 0.03). In ACC, methylation density of seladin-1 promoter was higher (2682 ± 686) than in normal adrenal glands (362 ± 97; p = 0.02). Seladin-1 mRNA expression in ACC (1452 ± 196) was significantly lower than in normal adrenal glands (3614 ± 949; p = 0.01).

**Conclusion:**

On this basis, methylation could be involved in the altered pattern of seladin-1 gene expression in ACC.

## Background

Seladin-1 (Selective Alzheimer disease indicator 1, seladin-1) gene (chromosome 1p33-31.1), firstly identified in neuronal cells [1], codes for the cholesterol synthesizing enzyme (3-beta-hydroxysterol delta-24-reductase, DHCR24) and show high homology to the Diminuto like protein, a cell elongation factor described in Arabidopsis thaliana [2]. The expression of seladin-1 mRNA and protein is down-regulated in brain regions affected by Alzheimer's disease [1,3].

The involvement of seladin-1 in human cancers was recently investigated. Seladin-1 mRNA expression is activated in response to Ras expression, showing features of a potential tumor suppressor involved in the oncogenic signalling mediated by Ras/p53 [4]. The ablation of this gene in the presence of oncogenic and oxidative stress results in cell transformation. With regard to the enzymatic activity of seladin-1 involving cholesterol synthesis, it is known that some cancers show increased cholesterol content compared to normal tissues. The latter finding was accompanied by an enhanced anti-oxidant activity and consequent resistance to oxidative stress, thus conferring selective growth advantage to tumor cells [5,6]. The inhibition of caspase 3 and a key role in cholesterol synthesis represent the two main biological functions of seladin-1. In the latter case, it is still unclear whether the effects of seladin-1 are an indirect consequence of the modulation of intracellular cholesterol, which has well documented protective effects *in vitro *and *in vivo *[7-11].

Seladin-1 was investigated in prostate [12-14], ovary [15], bladder [16] and breast cancer [17], in melanoma [18] and pituitary adenomas [19].

Adrenal gland is the human tissue with the highest levels of expression of seladin-1 [1] whereas mRNA levels are markedly reduced in adrenal carcinomas, reaching the lowest levels in advanced disease (stages III-IV) [20]. Nevertheless, the demonstrated antiapoptotic role of seladin-1 [4,21] mainly due to its H_2_O_2 _scavenging activity [22] may give rise to difficulties in the meaning of seladin-1 downregulation in adrenal cancer. However, increased proliferation rate in tumoral cells may lead to increased apoptotic death [20]. It has been also reported that seladin-1 mRNA expression in cultured cells is modulated by ACTH [20,23], suggesting that its down-regulation in adrenal cancer may be due to a reduced expression of its receptor, as a marker of loss of differentiation.

In the hypothesis that epigenetic modifications may influence differential expression of seladin-1 in adrenal cancer we analyzed whether methylation could be regarded as a mechanism of seladin-1 regulation in human adrenal cancer cell lines and tissues.

## Methods

### Cell cultures and treatment

Two adrenal gland-derived cell lines, H295R and SW13, were used for this study. Cell lines were maintained at 37°C in a 95% air and 5% CO_2 _fully humidified environment in a culture medium consisting of a 1:1 (vol/vol) mixture of DMEM/F-12 with 10% FBS, 2 mm glutamine, 100 U/ml penicillin, and 100 μg/ml streptomycin. For H295R, medium was enriched with a mixture of insulin/transferrin/selenium. Cells were plated in duplicate and treated with 5 μM of 5-Aza-2-deoxycytidine (5-Aza) for 1, 3 and 6 days. Three separate experiments were performed in the same experimental conditions and results were calculated as the mean of such replicates. RNA extraction was performed with Rneasy MiniKit Qiagen^® ^columns, while DNA was obtained by using QIAamp DNA Mini Kit (Qiagen, Milan, Italy).

### Patients

A total of 14 samples, collected in the Surgical Department of the Azienda Ospedaliera Careggi, Florence, with the approval of the local Ethics Committee, included 9 adrenal carcinomas and 5 normal adrenal from patients undergoing nephrectomy for renal cancer. Informed consent was obtained from each patient. All samples were accurately examined by an expert pathologist to prevent contaminations from contiguous tissues. Samples were immediately snap frozen in liquid nitrogen until acid nucleic extraction. For RNA extraction, tissues were disrupted by homogenisation in 600 μl of guanidine isothiocyanate containing lysis buffer and then processed with Rneasy MiniKit Qiagen^® ^columns. Genomic DNA was extracted from neoplastic tissues using DNeasy Tissue Kit (Qiagen Milan, Italy).

### Reverse transcription and quantitative real-time PCR

Total RNA (200 ng) was reverse transcribed in a 40 μl reaction mixture containing TaqMan RT buffer 1×, 5.5 mM MgCl2, 500 μM each dNTPs, 2.5 μM random hexamers, 0.4 U/μl RNase inhibitors and 1.25 U/μl MultiScribe reverse transcriptase. The profile of the one step reverse transcription reaction was 10 min at 25°C, 30 min at 48°C and 2 min at 95°C. The PCR reaction was performed with 25 ng cDNA, in a reaction mix containing 300 nM of forward primer, 900 nM of reverse primer, 12.5 μl Universal Master Mix and 200 nM of each fluorescent probe. Plates were treated 2 min at 50°C, 10 min at 95°C and then submitted to 40 cycles of amplification at 95°C for 15 s, 60°C for 60 s in the ABI Prism 7700 Sequence Detector PE Applied Biosystems (Foster City, CA, USA). Seladin-1 mRNA expression was evaluated with relative quantitative RT-PCR using primers and probe as previously reported [20]. The integrity of total RNA was verified in all samples with Agilent 2100 bioanalyzer and as reference gene the evaluation of GAPDH mRNA expression was performed by using the Pre-Developed TaqMan Assay Reagent, GAPDH endogenous control kit from Applied Biosystems.

### Methylation specific PCR

**Figure 1:**
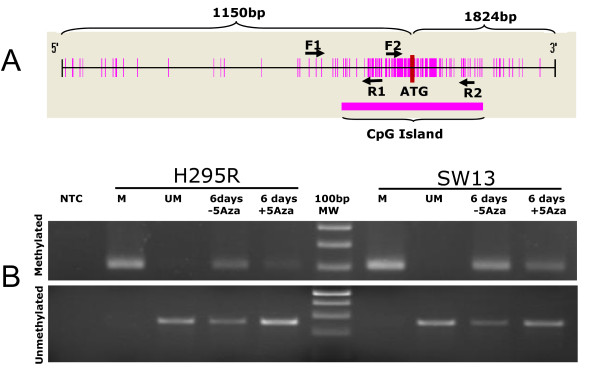
**Schematic representation of CpG island in seladin-1 promoter and methylation status in H295R and SW13 cell lines**. (Panel A) The large CpG island includes the translation start codon (ATG), spanning from -868 bp to +918 bp. The arrows F1 and R1 indicate primers which did not evidence any methylation sensitive sites. F2 and R2 are primers used for MSP and Q-MSP analysis. (Panel B) Methylation specific PCR performed in H295R and SW13 cell lines. Primers for methylated (upper gel) and unmethylated DNA (lower gel) recognized in both cell lines corresponding methylated and unmethylated seladin-1 promoter sequences. After 6 days of treatment with 5-Aza (+5Aza) we observed a reduction of methylated form in both cell lines in comparison to controls (-5Aza). Conversely, the intensity of bands corresponding to unmethylated DNA increased in intensity, confirming the demethylation of corresponding sequences. (NTC = no template control, MW = molecular weight marker, M = cell line methylated DNA, UM = cell line unmethylated DNA).

Methyl Primer Express^® ^Software v1.0 (Applied Biosystems, Foster City, CA, USA) was used to evaluate methylation-sensitive sites in seladin-1 sequence from -4384 bp and +1826 bp. The region from -4384 bp to -1150 did not reveal any CpG island. In the remaining sequence, we identified a large 1786 bp long CpG island from -868 bp o +918 bp. We analyzed two separate sequences in this region by MSP: the first, from -404 bp to -136 bp did not evidence methylation sensitive site (data not shown). In the second region, partially comprising exon1 and the transcription starting site (Figure 1; see also Ref [4] for more sequence details) we selected a primer set for methylated form with the following sequences: F2 meth 5'-CGGGTTGTGGGTTATAGGC-3', localised at -97 and R2 meth 5'-ACGAACACCCAACGCTAATAAAT-3' at +81 nucleotide from the same site (amplicon length 202 bp). The unmethylated form of the same sequence was amplified using the primers: F2unmeth 5'-TTGTGGGTTATAGGTGTAGAGT-3' at -93 nucleotide from translation start site and R2unmeth 5'-CCAAACACACACATAATAATAAA-3' at +141 (amplicon length of 258 bp).

DNA from cell lines and tissue samples (500 ng) was submitted to bisulphite modification using EpiTect Bisulfite Kit (Qiagen, Milan, Italy) following manufacturer's protocol. For each treatment, CpG Genome Universal Methylated and Unmethylated DNA (Chemicon International Inc, USA) were used as positive and negative controls to confirm specificity of methylation specific PCR (MSP). After bisulphite treatment, DNA was immediately submitted to PCR analysis.

MSP was performed submitting modified DNA to 94°C for 5 min and 50 cycles at the sequent conditions: 94°C for 30 sec, 58°C for 30 sec, 72°C for 30 sec and a final hold to 72°C for 10 min. PCR product were then resolved in a 2% agarose gel.

### Quantitative methylation specific PCR for methylation analysis

Q-MSP was performed to confirm qualitative results using a real time RT-PCR assay. Quantitative analysis was performed on RotorGene™3000 (Corbett Research, Australia) in a final volume of 10 μl with Quantitect Probe PCR Master Mix 2× (Qiagen, Milan, Italy), primers 600 nmol/l and probe 200 nmol/l and 1 μl of modified DNA. Specificity of the assay was achieved using the same primers for the methylated DNA together with an internal probe selected by PrimerExpress software (Applied Biosystems, Foster City, CA, USA) with sequence: 5'-TTG GCG GTA GTG ATA G-3', localised at -56 nucleotide from transcription start site and labelled with FAM. PCR conditions were: 95°C for 15 min, 55 cycles at 95°C for 15 sec and 60°C for 60 sec. In all samples β-Actin DNA was analysed after bisulfite treatment using primers and probe previously described [24] to perform relative quantification. As for mRNA analysis, changes in DNA methylation pattern were evaluated considering the treated/untreated ratio. The same protocol was used to perform seladin-1 quantification in normal adrenal gland samples and carcinomas.

### Pyrosequencing analysis

Analysis of sequences after bisulfite treatment was performed by Pyrosequencing™ (PyroMark ID System, Biotage, Sweden) technology. Primers selected to perform amplification were designed using PyroQ-CpG™ Software (Biotage, Sweden) and were: FW 5'-GTT TGA AGG GGT TGG AGT-3' and RV 5'-CTC ACC TAC TTC TAA ATA TCC C-3'. Amplification has been performed using one hundred nanograms of total DNA in a PCR reaction mix in 50 μl final volume. Samples were denatured for 9 min at 94°C followed by 40 cycles of amplification at 94°C for 1 min, 55°C for 1 min and then 72°C for 90 sec, in a Gene Amp 9700 Thermal Cycler (Applied Biosystems, Milan, Italy). Thirty μl of PCR product were used for immobilization by streptavidin sepharose beads (Streptavidin Sepharose™ High Performance, GE Healthcare Bio-Science AB, Sweden) and then incubation at 95°C for 5 minutes of single-stranded DNA with the sequencing primer, a biotynilated reverse primer with the same sequence used in PCR, has been performed.

### Statistical analysis

Statistical analysis was carried out using the SPSS software package (SPSS INC, Chicago, IL). The Wilcoxon Rank-Sum test for paired samples was used to test differences in control cells versus cells treated with demethylating 5-Aza, while Kolmogorov-Smirnov test data was used to evaluate differences between adrenal carcinomas and normal adrenal glands. Differences with p < 0.05 were considered statistically significant.

## Results

### Methylation specific PCR (MSP) of methylation-sensitive promoter region

DNA extracted from H295R and SW13 cell lines was submitted to MSP with primers targeting methylated and unmethylated alleles, alternatively. As reported in Figure 1, in both cases amplification generated amplicons with the expected size, suggesting that methylation of seladin-1 promoter is in hemi-methylated status in both cell lines. When cell lines were submitted to 5-Aza treatment, MSP revealed a reduction of the intensity of the band corresponding to methylated DNA in comparison to untreated cell lines, which was paralleled by an increase of amplification products when primers for unmethylated DNA were used (Figure 1, Panel B).

### Quantitative methylation specific PCR (qMSP) of seladin-1 promoter methylation in vitro

**Figure 2:**
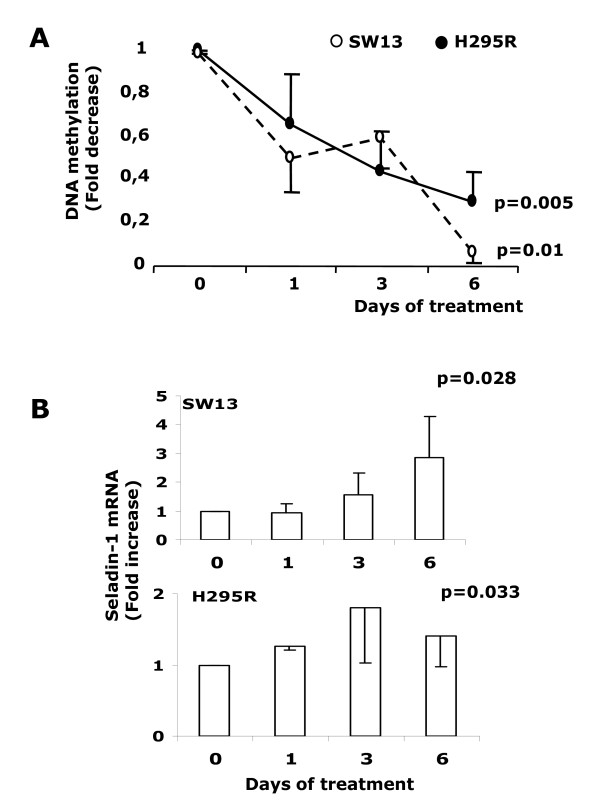
**Effects of 5 μM 5-Aza treatment on H295R and SW13cell lines**. (Panel A) Decrease of seladin-1 promoter methylation in DNA from the two cell lines. Results were expressed as fold decrease in comparison to T0 values. (Panel B) Increase of seladin-1 mRNA expression in the same cell lines after treatment with 5-Aza. Results were expressed as fold increase in comparison to T0 values.

We assessed the methylation and expression patterns of seladin-1 in adrenal cell lines submitted to the same protocol of treatment with 5-Aza. Results obtained from qMSP in DNA extracted from treated cell lines (Figure 2, panel A) indicated that the levels of DNA methylation progressively decreased in both cell lines under treatment, with the maximal effect of 5-Aza on DNA methylation observed after six days. Apparently the effect was more evident on SW13, probably due to the higher proliferation rate of this line in comparison to H295R cells. The same treatment induced a progressive up-regulation of seladin-1 mRNA expression. Once again the positive effect appeared more evident for SW13 cells, whereas the induction on seladin-1 expression in H295R cells was less marked (Figure 2, panel B).

### Relationship between seladin mRNA expression and methylation pattern in adrenal tissues

**Figure 3:**
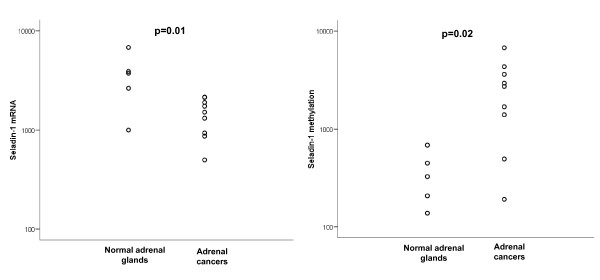
**Seladin-1 mRNA expression (panel A) and DNA methylation (panel B) in normal adrenal glands (n = 5) and carcinomas (n = 9)**.

To test whether seladin-1 could be epigenetically regulated also 'in vivo', promoter methylation and its mRNA expression were measured in 9 adrenal carcinomas and in 5 normal adrenal glands. For seladin-1 mRNA, our results confirmed those previously reported [20]: in adrenal carcinomas seladin-1 mRNA (1434 ± 192) was significantly lower than in normal adrenal glands (3433 ± 826; p = 0.01) (Figure 3). Conversely, DNA methylation evidenced a statistically significant (p = 0.02) increase in carcinoma samples (2633 ± 707) compared to normal tissues (362 ± 97).

**Figure 4:**
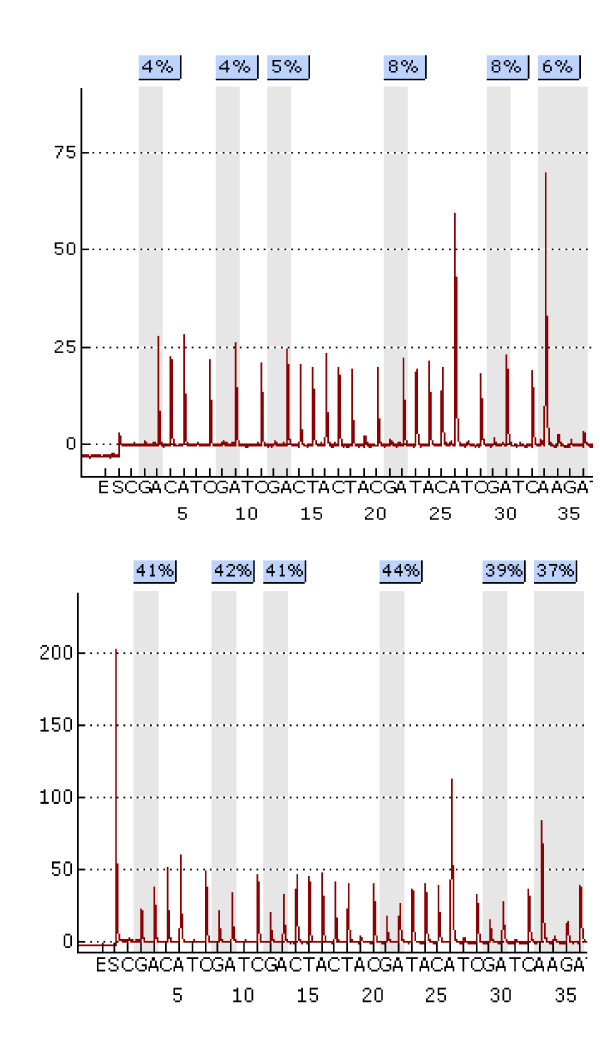
**Examples of two pyrograms showing a different pattern of methylation in one normal adrenal gland (upper panel) and one adrenal carcinoma (lower panel)**.

The presence of differential patterns of methylation between DNA from adrenal carcinomas and normal adrenal glands was confirmed with pyrosequencing. An example of respective pyrograms is reported in Figure 4.

## Discussion

Epigenetic events, such as DNA methylation, are crucial in establishing the correct pattern of gene expression. Disruption of this program leads to an aberrant mRNA transcription and potential loss of anti-cancer checkpoints.

The role of seladin-1 in cancer is still unclear, probably due to the multiple roles of this gene in regulating cell functions. However, a thorough analysis of seladin-1 role in oncogenesis and oxidative stress indicated that its expression is involved in the regulation of Ras-induced transformation and senescence in human and rodent cells [4]. Apparently, seladin-1 exerts its protective effects against oxidative stress following two independent ways. Seladin-1 is up-regulated as a response to acute oxidative stress, with a cholesterol-dependent mechanism, but is down-regulated upon chronic exposure to oxidative stress. However, also the reduction of seladin-1 expression appears involved in a prosurvival strategy due to its interaction with p53 status and function, as recently demonstrated [25]. The initial consideration that adrenal glands show very high mRNA expression levels of seladin-1 [1] and that adrenal carcinomas show a significant reduction of seladin-1 mRNA [20], has prompted a deeper analysis of this gene expression in adrenal cancer. On this basis, the definition of the regulation of seladin-1 expression seems to be crucial for the comprehension of the mechanisms underlying its downregulation in tumoral tissues.

In the present study we demonstrated for the first time the presence of a functionally active CpG island in the regulatory sequence of Seladin-1 gene. Qualitative and quantitative methylation specific PCR clearly indicated that in the adrenal cancer cell lines H295R and SW13 the CpG island is densely methylated and that the treatment with the 5-Aza was able to decrease DNA methylation. In the same experiments we demonstrated that the expression of seladin-1 mRNA could be directly related to the altered pattern of promoter methylation since exposure of adrenal cell lines to 5-Aza was associated to a significant induction of Seladin-1 mRNA expression in SW13 and H295R lines, even if the effects of 5-AZA on H295R methylation is apparently less evident than in SW13. This difference could refer to the different proliferation rate of the two cell lines. The higher proliferative rate of SW13 can explain the major effect of 5-AZA in reducing DNA methylation, since the incorporation of citosine analogue is much more elevated in cells with a shorter duplication time. After 6 days of treatment, methylation in SW13 is close to 0% and the effect of Seladin-1 mRNA expression is maximal. Conversely, H295R tend to proliferate more slowly and after 6 days 30% of their DNA is still methylated and the gain of mRNA expression, even if significantly increased, is less intense than in SW13.

In addition, we experienced also a different effect of the demethylating agent on the respective proliferation rate. 5-Aza treatment induced a reduction of cell growth in H295R that reaches a 50% inhibition after a 6-days treatment, as previously reported [26,27], whereas the reduction of cell line growth in SW13 was only 15% in comparison to controls. Therefore, the discrepancy of the effects in the two cell lines is probably connected to their ability to grow in normal cultural conditions and under 5-AZA treatment.

After these preliminary indications obtained 'in vitro', we tried to confirm the presence of an epigenetic control of seladin-1 expression also in 'ex vivo' samples. Real time analysis performed on the same promoter region in DNA of adrenal carcinomas, adenomas and normal glands showed an inverse relationship between methylation of seladin-1 and its expression. In particular hypermethylation was associated to reduced seladin-1 expression levels in adrenal cancer compared to normal adrenal gland and adenomas. No significant difference between adenomas and normal adrenal glands was evidenced. Thus, at least in adrenal carcinomas, hypermethylation could account for the reduction of mRNA expression [20].

To our knowledge, this is the first evidence that the pattern of expression of seladin-1 may be regulated by a differential methylation status of the promoter region of this gene, even if the transcription activation after 5-Aza is not a final confirmation of a direct epigenetic regulation on the gene itself. In fact an indirect effect resulting from the demethylation of other genes regulating seladin-1 expression cannot be excluded. Additional studies should be performed in order to determine whether the degree of methylation may account for the different levels of expression between normal and pathologic tissues detected in other human organs

## Conclusion

According to our results, methylation could be involved in the altered pattern of seladin-1 gene expression in ACC.

## Competing interests

The authors declare that they have no competing interests.

## Authors' contributions

LS carried out the molecular genetics study, the statistical analysis and drafted the manuscript. FM carried out cell line experiments and participated in results analysis. PL and CD carried out mRNA expression experiments. SG participated in the molecular genetics study and mRNA expression data analysis. RA participated in the molecular genetics study. MM and AP participated in the study design, results analysis and interpretations. CO conceived of the study, drafted the manuscript and participated in its design, coordination and results interpretations. All Authors read and approved the final version of the manuscript.

## Pre-publication history

The pre-publication history for this paper can be accessed here:

http://www.biomedcentral.com/1471-2407/10/201/prepub
